# Challenges and opportunities for alkane functionalisation using molecular catalysts

**DOI:** 10.1039/c7sc03610h

**Published:** 2017-11-09

**Authors:** Xinxin Tang, Xiangqing Jia, Zheng Huang

**Affiliations:** a State Key Laboratory of Organometallic Chemistry , Shanghai Institute of Organic Chemistry , University of Chinese Academy of Sciences , Chinese Academy of Sciences , 345 Lingling Road , Shanghai 200032 , China . Email: huangzh@sioc.ac.cn

## Abstract

The conversion of vast low-value saturated hydrocarbons into valuable chemicals is of great interest. Thanks to the progression of organometallic and coordination chemistry, transition metal catalysed C sp^3^–H bond functionalisation has now become a powerful tool for alkane transformations. Specifically, methods for alkane functionalisation include radical initiated C–H functionalisation, carbene/nitrene insertion, and transition metal catalysed C–H bond activation. This perspective provides a systematic and concise overview of each protocol, highlighting the factors that govern regioselectivity in these reactions. The challenges of the existing catalytic tactics and future directions for catalyst development in this field will be presented.

## Abstract 2

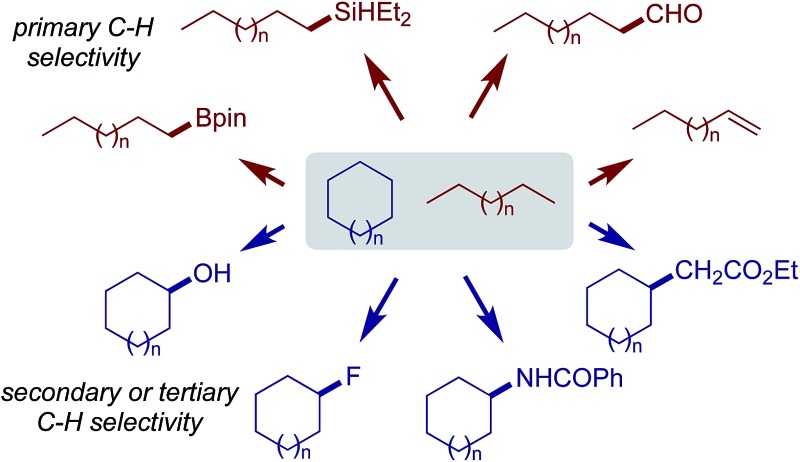
The conversion of vast low-value saturated hydrocarbons into valuable chemicals is of great interest.

## 1. Introduction

Alkanes, or paraffins, with the general chemical formula of C_*n*_H_2*n*+2_, have the simplest structure among organic molecules, which range in complexity from the smallest hydrocarbon, methane, to macromolecules such as polyethylene. Alkanes are the major constituents of natural gas and crude oil and thus the direct conversion of abundant and low-cost hydrocarbon feedstocks into value-added products has the potential to be a revolutionary technology in the chemical industry. Selectively converting propane to propene, for instance, is a process with tremendous applicability due to the huge demand of propene for the production of polypropene and commodity chemicals, such as acrylic acid, acrylonitrile, and cumene.

**Fig. 1:**
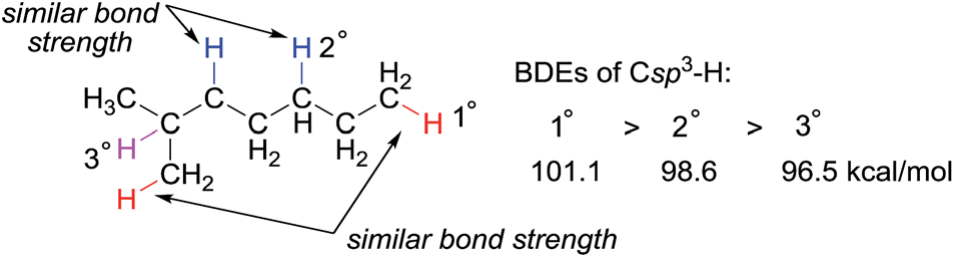
Bond dissociation energies (BDEs) of primary, secondary, and tertiary C sp^3^–H bonds in an alkane.

Alkanes are among the least reactive organic molecules as they consist only of strong C sp^3^–H and single C sp^3^–C sp^3^ bonds. The chemical inertness of alkanes is somewhat reflected in the forcing conditions that are required in the very few alkane transformations performed industrially: (hydro) cracking or reforming processes in oil refineries using heterogeneous catalysts operate at temperatures in the range of 400 to 600 °C. Another general concern for alkane functionalisation is the control of regioselectivity. In the absence of a directing or activating group, the reactivity of one C–H or C–C bond in an alkane is expected to be similar to that in the others.^1^ Taking C–H bonds as an example, the bond dissociation energies (BDEs) of the primary (1°), secondary (2°) and tertiary (3°) C sp^3^–H bonds of alkanes fall in the range of 96 to 101 kcal mol^–1^, with 1° C–H bonds being stronger than 2° and 3° bonds.^2^ The BDE values are close, which renders the discrimination of two C sp^3^–H bonds, in particular the same type of bonds, very difficult (Fig. 1). Finally, the product of alkane functionalisation is generally more reactive than the starting material. Hence, the transformation will be subject to an upper limit on the yield of the end product.

Developing homogeneous catalysts for alkane functionalisations has garnered increasing attention during the past four decades due to the potential advantages of milder conditions, higher selectivity, and an enhanced diversity of transformation modes compared to heterogeneous catalysis. While many methods using small-molecule catalysts have been developed for the selective functionalisation of a specific C sp^3^–H bond, examples of C–C bond activation in simple alkanes are rare.^3^ Therefore, catalytic alkane transformations have greatly benefited from the spectacular progress in the field of C–H bond functionalisation seen over the last several decades. The reactions fall under three general categories: (i) stepwise radical pathways, (ii) carbene/nitrene insertion, and (iii) metal-mediated C–H bond activation. It is important to note that, depending on the pathways through which the C–H bond is cleaved, the relative reactivity of different types of C–H bond varies. Although 1° C–H bonds are less reactive towards a free radical or metallocarbene than 2° and 3° C–H bonds, many organometallic reagents and catalysts react preferentially with 1° C–H bonds over 2° and 3° C–H bonds. This perspective will provide representative examples for each class of alkane functionalisation with an emphasis on the selectivity for different types of C sp^3^–H bond, highlight the inherent challenges for these catalytic reactions and outline future prospects in this area. Although it is very important, methane functionalisation is beyond the scope of this perspective and heterogeneous and enzymatic systems will not be covered here either.

## 2. Radical-induced alkane functionalisation

**Fig. 2:**
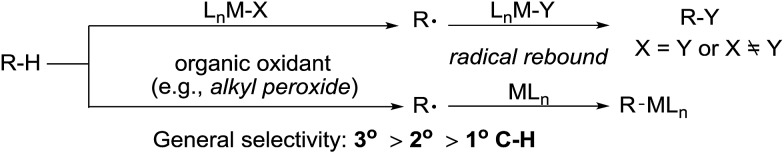
Alkane functionalisation through a radical intermediate.

Alkane functionalisation can occur through hydrogen abstraction from a C–H bond by either a high valent metal-based radical (*e.g.* porphyrin oxoiron(iv) cation radical) or an organic oxidant (*e.g.* alkyl peroxide) to form an alkyl radical, which is trapped by a metal complex to form the functionalised product or rebound to a metal centre to form a metal-alkyl species that is subject to further transformation (Fig. 2). The past 40 years have seen significant advances in the development of enzymatic catalysis for alkane oxidations and the reader is directed to several recent reviews for an in-depth coverage.^4^ Meanwhile, many synthetic metalloporphyrin and non-heme metal complexes have been developed for alkane hydroxylations, halogenations, and other transformations.^5^ The selectivity of these reactions in general correlates with the C–H bond strength.^6^ However, when a sterically demanding metal complex is involved in the recombination with an alkyl radical, the reaction tends to occur at the 2° and 1° C–H bonds, rather than at the 3° C–H bonds, as demonstrated by one example shown below.^7^,^8^

In 1979, Groves reported the first example of the porphyrin Fe-catalysed hydroxylation of C sp^3^–H bonds with iodosylbenzene as the oxidant. The oxidation of adamantane using [Fe^III^(TPP)Cl] (TPP = tetraphenylporphyrin) gives low yields of alcohols. The product distribution reveals the selective hydroxylation of 3° C–H over 2° C–H bonds (eqn (1)).^9^ Importantly, an oxoiron(iv) porphyrin cation radical was well-characterized by NMR, EXAFS and Mössbauer spectroscopy, thereby providing evidence in support of a radical mechanism.^10^
1

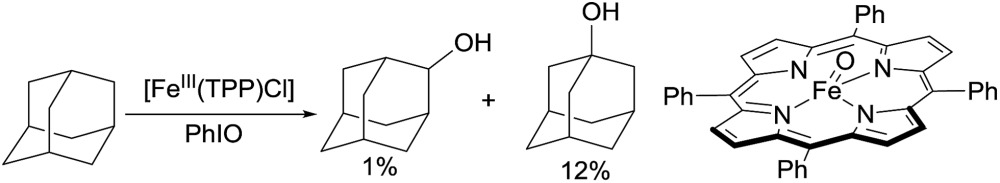



2

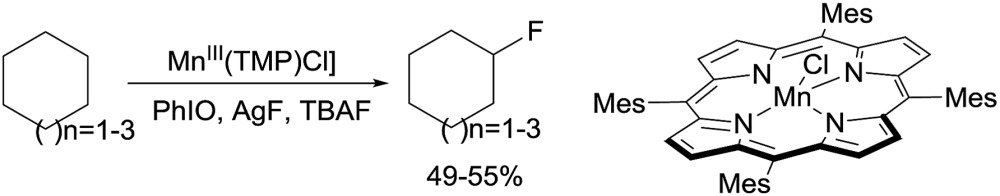




Following their early work on C–H bond hydroxylation, recently the same group reported the halogenation of alkanes catalysed by porphyrin Mn complexes through a so-called heteroatom-rebound catalysis strategy.5*c* Selective halogenation over common hydroxylation is due to the decelerated oxygen-rebound rate when the *trans*-axial ligand (L) bound to the Mn centre (L–Mn^V^=O) is a OH^–^ or F^–^ group. Brominations,^11^ chlorinations,^11^ and even fluorinations^12^ occur with moderate-to-high yields for cycloalkanes. For example, the fluorination of C6–C8 cycloalkanes with AgF/TBAF using Mn(TMP)Cl (TMP = tetramesitylporphyrin) affords the mono-fluorinated products in ∼50% yields (eqn (2)). More recently, the group extended the Mn catalysis to the azidation of 2° and 3° alkane C–H bonds using aqueous NaN_3_ as the azide source.^13^

Most of the radical reactions use cycloalkanes as the substrates, in part because the regioselectivity issue does not arise in such cases. In 2014, Che reported the oxidation of acyclic light alkanes, ethane and propane, with oxone as the oxidant and a non-heme Fe complex [Fe^III^(Me_3_tacn)(Cl-acac)Cl]^+^ (Me_3_tacn = 1,4,7-trimethyl-1,4,7-triazacyclononane) as the precatalyst.^14^ The reaction of propane gives a mixture of iso-propanol/acetone/propanoic acid in a 4 : 11 : 1 ratio, with a total turnover number (TON) of 20 (eqn (3)). The regioselectivity observed in the reactions is consistent with the selectivity of 2° > 1° for alkane oxidation by a radical pathway. Thorough mechanistic studies established an oxoiron(iv) radical intermediate, [Fe^IV^(Me_3_tacn)(Cl-acac˙^+^)(O)]^2+^.
3

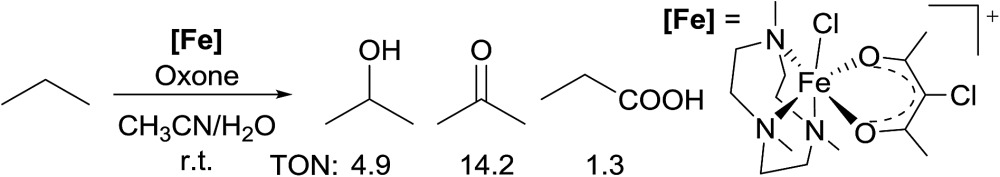




Alkane functionalisations with alkyl peroxides or hypervalent iodine compounds as the radical initiators have also been documented.^7^,^15^ In 2014, Hartwig reported Cu-catalysed amidations or imidations of alkanes with amides, sulfonamides, or imides in the presence of *t*BuOO*t*Bu (DTBP).^7^ Intriguingly, the regioselectivity of 2° > 1° > 3°, as demonstrated by the reaction of 3-ethylpentane (eqn (4)), differs from the regiochemistry observed for most radical-induced C–H bond functionalisations. The low reactivity of the 3° C–H bond in this case is attributed to steric effects: the tertiary radical is too sterically encumbered to recombine with phen-ligated Cu amidate, a proposed catalytic intermediate. More recently, Lei published an interesting example of alkane alkynylation through a radical oxidative C sp^3^–H/C sp–H cross-coupling using a mixed Cu/Ni/Ag catalytic system. The product distributions shown in eqn (5) indicate that the 2° C–H bonds are more reactive than the 1° C–H bonds for the coupling.15*c*
4

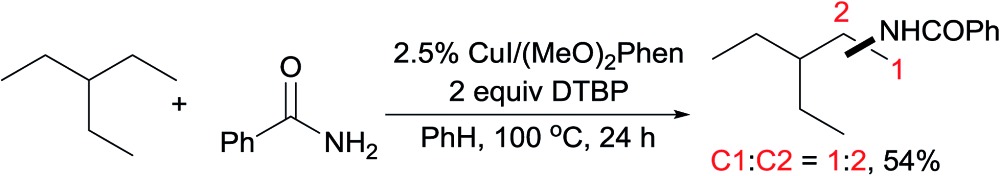



5

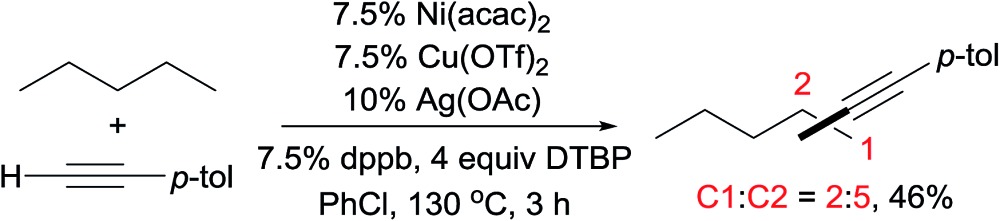




To date, a number of methods for alkane functionalisation *via* radical pathways have been reported^15^,^16^ but the substrate scopes are primarily limited to cycloalkanes. Catalytic functionalisations of linear alkanes invariably lead to mixtures of regioisomers, as exemplified above. In particular, the direct functionalisation of alkanes at the terminal positions by a radical mechanism remains unknown. Most recently, the Baudoin^17^ and Martin^18^ groups independently developed two-step protocols for the terminal functionalisation of alkanes. Such systems comprise a Mn-mediated alkane bromination with Br_2_, yielding a mixture of alkyl bromides and a subsequent Pd/Ni-catalysed tandem chain-walking and arylation/carboxylation sequence. These methods show high selectivity for linear products (eqn (6) and (7)).
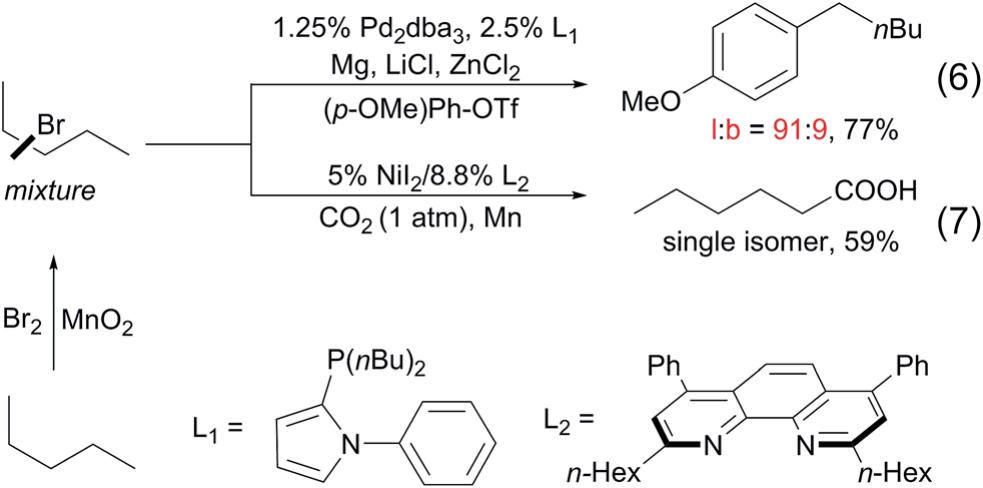


8

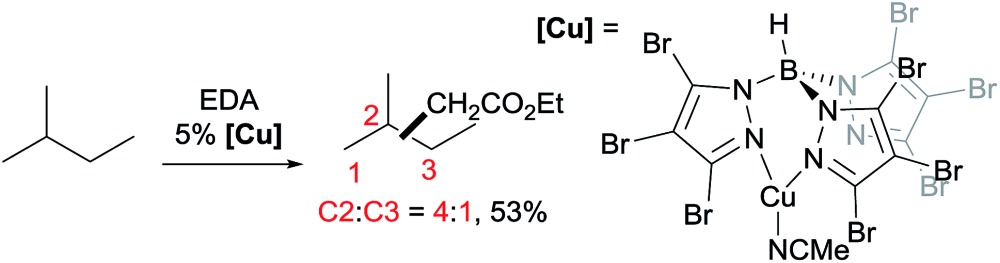




## 3. Alkane functionalisation by carbene or nitrene insertion

**Fig. 3:**
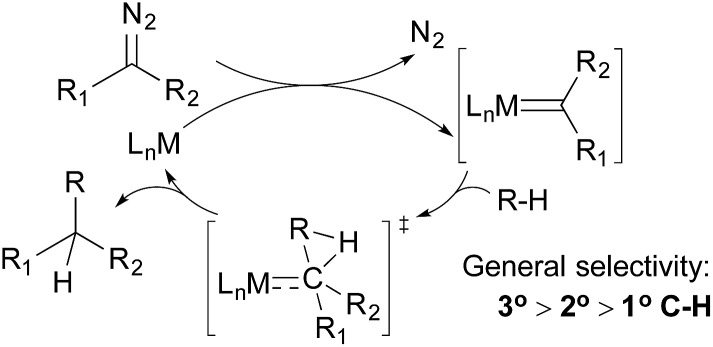
Alkane functionalisation *via* carbene insertion.

Alkane functionalisations by means of carbene insertion into C–H bonds occurs through a concerted pathway, involving a three-centered transition state formed by the overlap of an empty p-orbital of the carbene carbon with the C–H σ-orbital of the alkane (Fig. 3). Consequently, the product is formed with the retention of the configuration at the carbon centre.^19^ Like the radical mechanism shown above, the metal centre does not interact directly with the C–H bond, but the metal fragments have important effects on the stability, reactivity, and selectivity of the carbene moiety: (i) an appropriate transition metal can serve as a stabilizing entity, protecting the carbene from decomposition by forming a transient metallocarbene; (ii) a metal complex with an electron-withdrawing ligand can enhance the electrophilicity of the carbene, making it more reactive towards weak nucleophiles such as alkanes and (iii) the general regiochemical preference for carbene insertion into 3° over 2° C–H bonds and 2° over 1° C–H bonds is similar to that observed for the radical pathway, but the steric environment of the ligand may have a large impact on regiochemistry and even on stereochemistry.

In the 1970s, Scott and DeCicco disclosed the first example of carbene insertion into alkane C–H bonds using simple copper sulfate as the catalyst; the reaction of cyclohexane with ethyl diazoacetate (EDA) results in a low yield of the C–C bond-forming product.^20^ Years later, Noels and Teyssie reported an improved system using dirhodium catalysts with dicarboxylate ligands; the reactions of various cyclic and linear alkanes give moderate-to-good yields of C–H monoinsertion products.^21^ These seminal works set the stage for catalyst development in this field – most systems developed later use Rh- and coinage metal-based catalysts.

A series of contributions mainly from the Pérez group on coinage metal catalysis significantly advanced the area of alkane functionalisation *via* carbene insertion.^22^–^25^ A Cu complex of electron-withdrawing perbromo trispyrazolylborate catalyses carbene insertion into the C–H bonds of linear alkanes with moderate to good yields and a regioselectivity of 3° > 2° > 1° C–H bonds (eqn (8)).^23^ While the Cu complex is hardly active for 1° C–H bonds,^23^,^24^ Ag24b,^25^ and Au24*a* analogs show enhanced primary selectivity. Among them, a highly electrophilic Ag complex of perfluoro trisindazolylborate gives 47% primary selectivity in the reaction of *n*-hexane with EDA (eqn (9)).25*b*
9

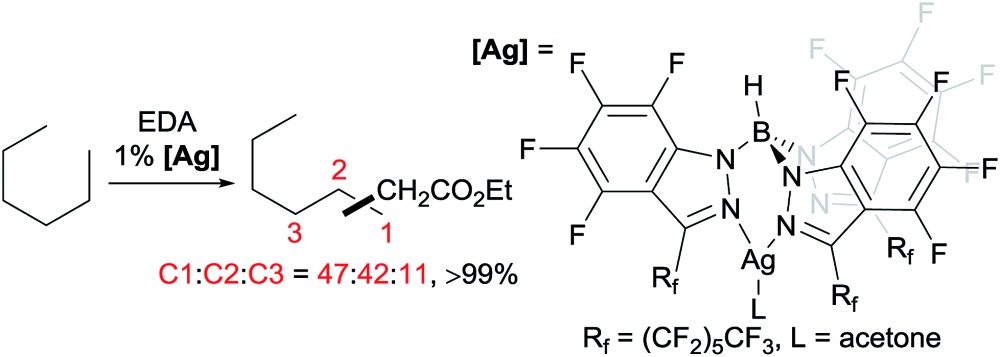



10

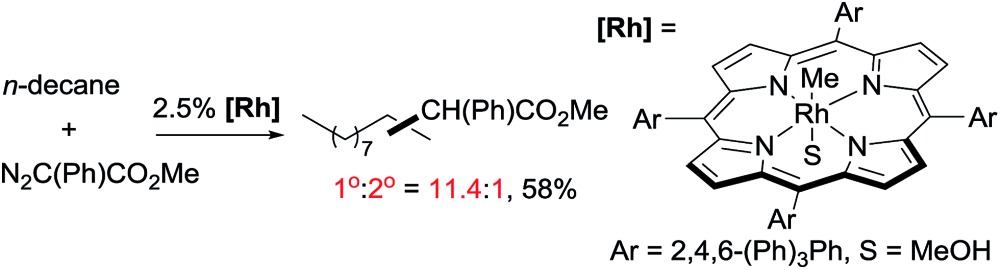




As mentioned above, the selective carbene insertion into a primary C–H bond is a challenge. Thus far the best terminal selectivity was achieved by Che using a Rh catalyst with a highly sterically demanding porphyrin ligand and a relatively bulky carbene source, N_2_C(Ph)CO_2_Me. For example, the reaction of *n*-decane offers ∼90% primary selectivity (eqn (10)).^26^ The steric contribution, evidently, prevails over the electronic effect in controlling the regioselectivity in this case.
11

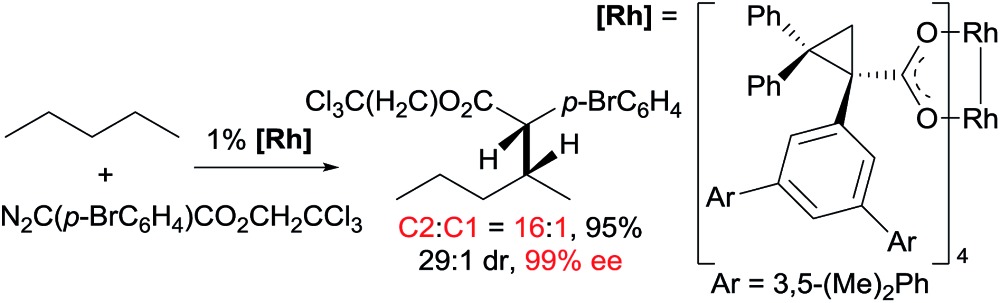




Asymmetric carbene insertion into alkane C–H bonds has also been achieved. The Davies group disclosed that dirhodium tetrakis (*S*-(*N*-dodecylbenzenesulfonyl)prolinate) could induce the asymmetric functionalisation of cycloalkanes with phenyldiazoacetate with modest-to-high enantioselectivity.^27^ Che extended the substrates to linear alkanes: the reaction of *n*-hexane with a sterically hindered chiral rhodium porphyrin is moderately selective for 1° C–H bonds (78% terminal selectivity), giving the linear product with 66% ee.^26^ A more recent example by Davies showed that the reaction of *n*-pentane and trichloroethyl 2-(4-bromophenyl)-2-diazoacetate using a dirhodium catalyst occurs selectively at the C2-position, accompanied with exceptionally high enantio- and diastereoselectivity (eqn (11)).^28^
12

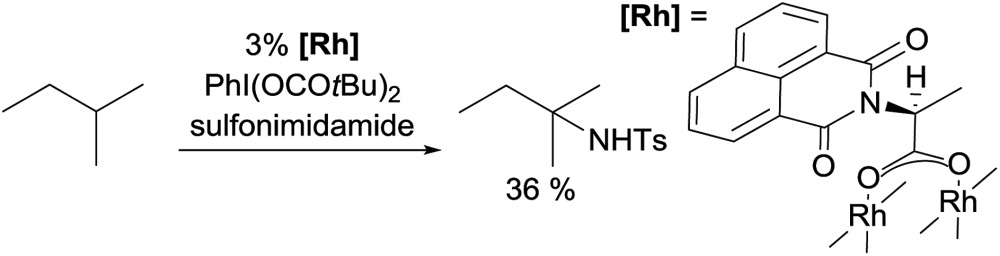




While carbene insertion reactions have been well documented, far less is known about nitrene insertions for alkane functionalisations. Early examples came from the Breslow^29^ and Barton^30^ groups using an Fe or Mn catalyst for aminations of cyclohexane and adamantane. Since then, several metal catalysts (Rh,^31^ Ag,31b,^32^ and Cu32b,^33^) have proven effective for the amination of cycloalkanes with sulfonylazide or hypervalent iodine as the nitrene source.

Examples of nitrene insertion reactions with acyclic alkanes are particularly scarce.32*a* The regioselectivity of 3° > 2° > 1° C–H bonds observed in the few reactions indicates that the product distributions are governed by the BDE of C–H bonds. For example, the Rh-catalysed reaction of 2-methybutane with sulfonimidamide is highly selective for tertiary C–H bonds, albeit in low yields (eqn (12)).31*c* The Ag-catalysed nitrene insertion into *n*-pentane gives a total yield of 65%, with branched amines as the major products (eqn (13)).32*c* Of note is that the asymmetric nitrene insertion into alkane C–H bonds has not been reported.
13

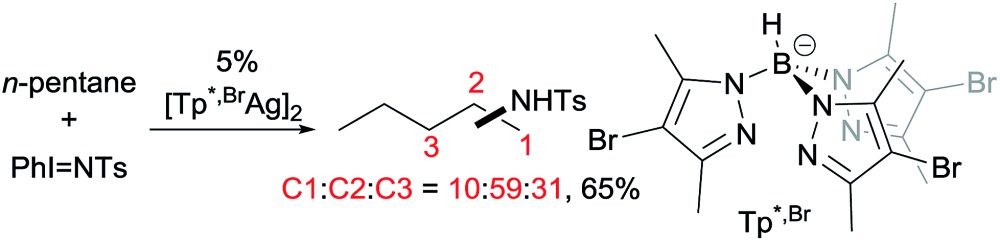



14

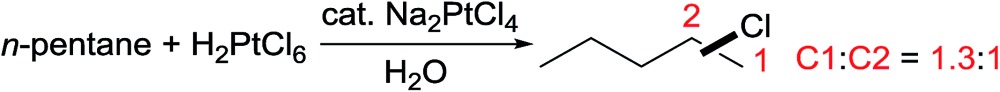




## 4. Alkane functionalisation *via* C–H bond activation at the transition metal centre

**Scheme 1:**
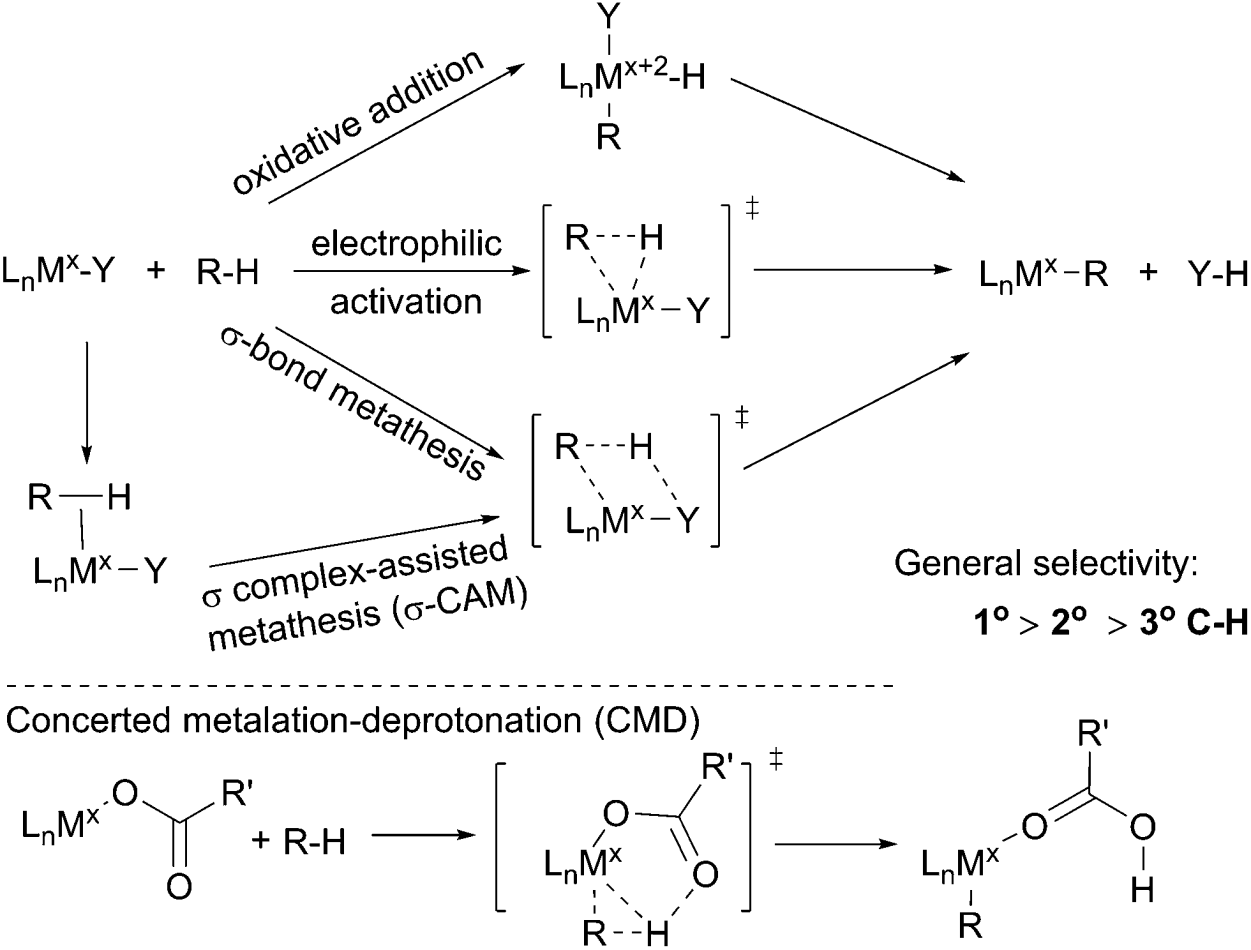
Pathways for metal-mediated C–H bond activation.

The term C–H bond activation refers to a type of reaction in which a transition metal reacts with a C–H bond to form a metal–carbon bond. Such transformations, traditionally, are classified into three categories: (i) oxidative addition with an electron-rich late transition metal;^34^ (ii) electrophilic activation with an electron-deficient late transition metal^35^ and (iii) σ-bond metathesis with an early transition metal *via* a four-membered transition state.^36^ Recently, the σ-bond metathesis mechanism has been extended to late transition metal systems involving σ complexes as intermediates, which was designated as σ complex-assisted metathesis (σ-CAM).^37^ Moreover, a variation of the electrophilic activation mechanism, termed concerted metalation–deprotonation (CMD), which often involves an electrophilic metal carboxylate, has been revealed recently (Scheme 1).^38^ Such routes by which metal–carboxylate complexes cleave C–H bonds, however, have been barely reported for the functionalisations of simple alkanes.

One attractive feature of metal-mediated C–H bond activation is the selective cleavage of the most sterically accessible primary C–H bonds. Bergman^39^ and Jones^40^ reported that Cp*M(PMe_3_) (M = Ir or Rh), generated upon the UV irradiation of Cp*Rh(PMe_3_)H_2_, reacts with an alkane to form Cp*M(PMe_3_)(*n*-alkyl)(H) with exclusive terminal regioselectivity. These proof-of-concept studies demonstrated the potential of C–H activation for the terminally selective functionalisation of an alkane. The known catalytic C–H bond functionalisations encompass alkane oxidation, borylation, carbonylation, dehydrogenation, and dehydrogenation based reactions, which will be presented in the following sections.

### 4.1. Alkane oxidation

In early 1970s, Shilov reported the selective partial oxidation of an alkane to an alcohol or alkyl chloride catalysed by PtCl_2_ using H_2_PtCl_6_ as the oxidant.^41^ The rate-determining step involves the electrophilic activation of R–H with a Pt(ii) centre to form a Pt(ii)–R complex. Next the Pt(ii)–R species is oxidized by [PtCl_6_]^2–^ to generate a Pt(iv)–R complex, which undergoes nucleophilic attack by OH^–^ or Cl^–^ with the release of the product and regeneration of the Pt(ii) catalyst.^42^

In principle, the expensive Pt(iv) oxidant could be replaced by a cheaper oxidant. Indeed, Cu(ii)/O_2_, Fe(iii)/O_2_ and polyoxometallate [H_3_PMo_9_V_3_O_40_]/O_2_ can act as effective oxidants for alkane oxidations.^43^ Periana discovered that SO_3_ or H_2_SO_4_ is an active oxidant for the catalytic oxidation of methane to methyl bisulfate in high yields. Most systems developed by this group were applied for methane oxidization;^44^ the reactions of higher alkanes were plagued by the formation of multiple products.
15

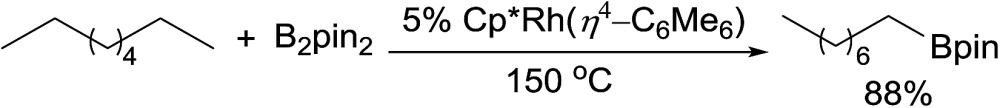




The selectivity for electrophilic C–H activation follows the trend of 1° > 2° > 3° C–H bonds, but the regioselectivity observed in the oxidation of an acyclic alkane (*e.g. n*-pentane in eqn (14)) is rather low.^45^ While the Pt-catalysed oxidations of alkyl sulfonates^46^ and ammonium salts^47^ offer moderate-to-good selectivity for primary C–H bond hydroxylation, to date, oxidations of simple alkanes with high terminal selectivity *via* electrophilic C–H activation have remained underexploited.43e,^48^

### 4.2. Alkane borylations

The catalytic alkane borylation developed by the Hartwig group is a unique example of the selective functionalisation of a primary C–H bond.^49^ The earlier systems with carbonyl complexes employed photochemical conditions. In 1997, Hartwig reported that a boryl W carbonyl complex, under irradiation, reacts with *n*-pentane to form a linear alkylboronate ester with excellent terminal selectivity.^50^ The stoichiometric reaction was then expanded to a catalytic reaction using a tricarbonyl Re catalyst.^51^ Under a CO atmosphere, the photocatalytic borylation of *n*-pentane with 1 equiv. of B_2_pin_2_ forms the linear product in high yield. Following the success of photocatalysis, this group developed methods for thermal alkane borylation. A Rh complex Cp*Rh(η^4^-C_6_Me_6_), upon thermally dissociating the dative ligand, hexamethylbenzene, proved effective for alkane borylation at 150 °C (eqn (15)).^52^ This catalytic system was also applied to the selective borylation of 1° C–H bonds in polyolefins.^49^,^53^ Subsequently, a simple Cp*Ru dichloride complex was found to be a suitable precatalyst for the borylation.^54^
16

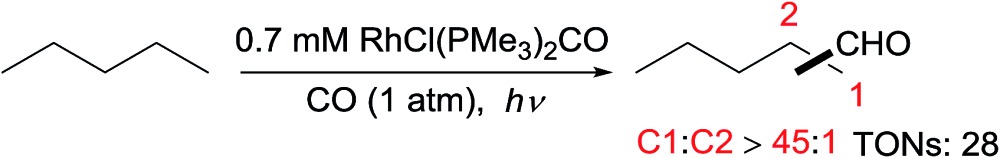




Calculations and experiments showed that the rate-limiting step in the Rh-catalysed borylation is the C–H bond activation,^50^–^52^ which very likely occurs through the σ-CAM route involving the interaction between a Rh–B bond and the coordinated C–H bond.^55^

### 4.3. Photocatalytic alkane carbonylation

Tanaka first reported alkane carbonylation with CO *via* C–H activation catalysed by RhCl(CO)(PMe_3_)_2_ under irradiation.^56^ The primary C–H bond of an acyclic alkane is selectively activated to form linear aldehydes, along with small amounts of branched products (eqn (16)). However, the resulting aldehyde could undergo a secondary Norrish Type II photoreaction to give a terminal olefin, acetaldehyde and ethanol as byproducts. The Goldman group revealed that this reaction occurs *via* at least two different mechanisms, one *via* a photochemical pathway and the other *via* a radical process. The former is responsible for the high linear selectivity, while the latter is less regioselective.^57^
17

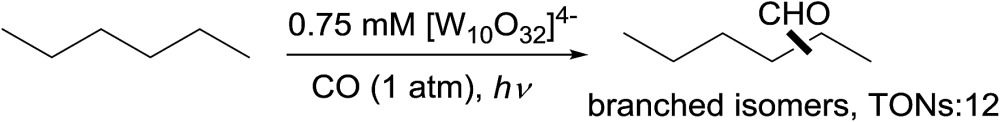



18

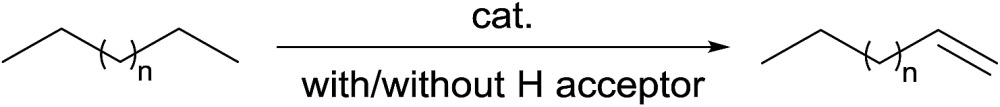




A pure radical process for alkane carbonylation was also reported – Hill showed that the polyoxotungstate-catalysed carbonylation of *n*-hexane with CO under irradiation produced a mixture of branched aldehydes (eqn (17)), in sharp contrast to the high linear selectivity gained in the rhodium catalysis.^58^ Note that catalytic thermal alkane carbonylation is an unknown reaction.

### 4.4. Alkane dehydrogenation

Alkane dehydrogenation (AD) is an atom-economic method for the synthesis of olefins, which are valuable synthetic intermediates for petrochemicals. Heterogeneous AD requires high reaction temperatures and suffers from low product selectivity.^59^ In this respect, the development of a mild and selective homogeneous AD system is desired. Pioneered by Crabtree and Felkin, significant advances have been achieved in the development of molecular catalysts for AD with or without a hydrogen acceptor (eqn (18)). In the majority of these systems Ir pincer complexes have been extensively studied, although the development of other metal systems has also received research interest.^60^ Most of these processes proceed *via* C–H oxidative addition and the formation of metal–carbon bonds tend to occur at 1° C–H bonds rather than 2° and 3° C–H bonds. Thus, organometallic AD is fundamentally promising for the production of α-olefins.
19

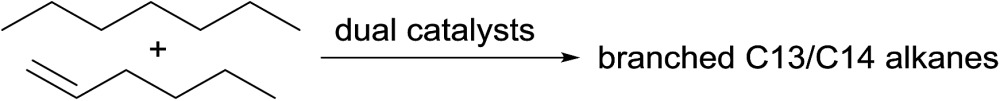




In 1979, Crabtree reported the first stoichiometric AD reaction: [IrH_2_(acetone)_2_(PR_3_)]^+^ reacts with cylcooctene (COE) to form cyclooctadiene Ir complexes with *tert*-butylethylene (TBE) as the hydrogen acceptor.^61^ Soon after that, the first examples of the catalytic AD transfer dehydrogenation (TD) of cylcooctane (COA) with TBE were independently developed by Crabtree^62^ and Felkin^63^ using Ir- or Re-based catalysts, albeit with low TONs. The hydrogenation of TBE overcomes the thermodynamic challenge associated with hydrogen release during the dehydrogenation process.

**Fig. 4:**
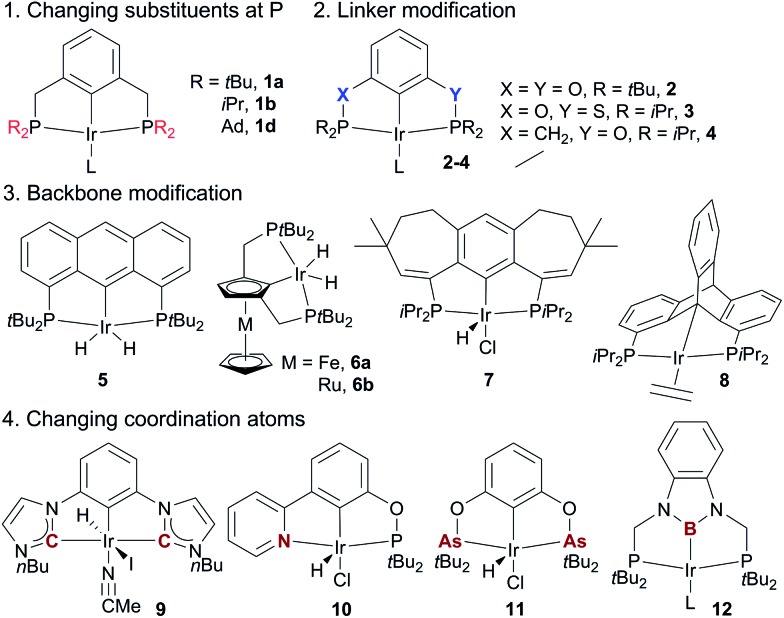
The design of various pincer Ir dehydrogenation catalysts.

A breakthrough in homogeneous AD was achieved in the mid-1990s by Jensen, Kaska, and Goldman, showing that bis(phosphine)-based (PCP)Ir pincer complexes are highly efficient for the TD of cyclic and linear alkanes. The success of this type of Ir system in AD is attributed in a large part to their high thermostability. The TD of COA/TBE using a *t*Bu-substituted complex (^*t*Bu4^PCP)IrH_2_**1a** (Fig. 4) at 200 °C gives an initial rate of 12 turnovers per minute. TONs of up to 1000 could be achieved by the addition of portions of TBE as the catalyst is subject to TBE inhibition at high [TBE].^64^

Catalyst **1a** is even effective for acceptorless dehydrogenation, giving 360 TONs in a run with cyclodecane at 201 °C after a day.^65^ The efficient hydrogen removal under refluxing conditions is crucial to the catalytic turnover. A sterically less hindered iPr-substituted analog (^iPr4^PCP)IrH_4_**1b** is even more effective than **1a**, producing a TON of 1000 for the acceptorless dehydrogenation of cyclodecane.^66^

Remarkably, Goldman demonstrated that catalysts **1a** and **1b** exhibit high kinetic selectivity for valuable α-olefins in the early stages of the TD of linear alkanes with TBE, NBE or α-olefin as the hydrogen acceptor. Unfortunately, subsequent olefin isomerization quickly leads to thermodynamically more stable internal olefins.^67^

The superior performance of (PCP)Ir complexes **1a** and **1b** in AD has stimulated extensive research in developing new pincer Ir complexes with the goal of more effective catalysts. Four strategies have been used for ligand design (Fig. 4). (1) Variation of substituents on the P-atoms:^68^ the *t*Bu-to-Me substitution, for example, leads to a more efficient AD catalyst (^*t*Bu3Me^PCP)IrH_4_**1c**.68*a* (2) Variation of the linkers between the backbone and two P-atoms: (^*t*Bu4^POCOP)Ir catalyst **2** containing two O-linkers^69^ and (^iPr4^PSCOP)Ir catalyst **3** with O and S as the linkers^70^ are less subject to TBE inhibition relative to the (^*t*Bu4^PCP)Ir system, producing TONs of up to 6000 in the reaction of COA/TBE TD. (3) Backbone modifications: complexes of various aromatic skeletons,^71^ including anthraphos **5**,71*a* ferrocene/ruthenocene **6**,71*b* 7-6-7 fused-ring backbones **7**,71*c* and triptycene **8**71*d* backbones, exhibit high thermostability and high activity for AD. (4) Substitution of coordination atom(s):^67^ bis(carbene) Ir (CCC, **9**),72a,b (PBP)Ir **12**,72*c* and pyridine–phosphinite (NCP, **10**)72*d* complexes display low-to-moderate activity in the COA/TBE TD. The novel (AsCAs)Ir complex **11** shows good TD activity, but decomposes readily at >175 °C.72*e*

**Fig. 5:**
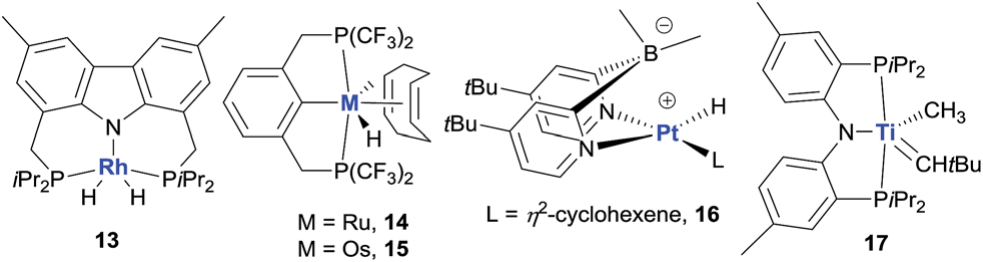
Non-iridium metal dehydrogenation catalysts.

Non-iridium precious metal catalysts have also been prepared and studied for AD, but in general they are less efficient than the classic pincer Ir complexes (Fig. 5). The studies of Rh catalysis mainly focused on photocatalytic AD (see below). One recent exception was the study by Brookhart using a carbazolide-based PNP Rh complex **13**, which affords moderate activity in the thermal TD of COA/TBE.^73^ Early investigations into Ru-catalysed dehydrogenation used Ru polyhydride63*b* and bis(phosphine) Ru(ii) bis(allyl) complexes,^74^ which showed low catalytic activity. A π-accepting pincer-ligated [^(CF_3_)_4_^PCP]RuH(COD) **14** gives a limited TON due to facile catalyst decomposition,^75^ while the Os analog **15** has a significantly enhanced lifetime relative to **14**, furnishing a TON of ∼600 in the COA/TBE TD.^76^ The author’s group developed a series of hydrido Ru(ii) olefin complexes supported by iPr-substituted pincer ligands, which are moderately active for the COA/TBE TD. Although less efficient, pincer Ru catalysts are more tolerant of polar functional groups than the related pincer Ir catalysts.^75^,^77^ A lipophilic *t*Bu_2_dpb-ligated hydrido Pt complex **16** shows low activity for the TD of cycloalkanes with TBE.^78^

Apart from the extensively studied noble metal-based AD catalysts, very recently, Mindiola and Baik reported a rare base-metal catalyst, (PNP)Ti alkylidene (**17**, Fig. 5), for TD with a phosphorus ylide as a hydrogen acceptor under mild reaction conditions (75 °C). Significantly, α-olefins could be selectively formed in this system, albeit with low TONs (<4).^79^

Compared to TBE, the most commonly used hydrogen acceptor for TD reactions, ethylene and propylene, are more practical acceptors in terms of cost and accessibility. Goldman and Brookhart reported the use of propylene as a hydrogen acceptor for the pincer-Ir-catalysed dehydroaromatization of *n*-alkane (*vide supra*).^80^ Furthermore, Brookhart demonstrated that *n*-pentane could be effectively dehydrogenated by pincer-Ir using ethylene or propylene as the acceptor; the resulting diene product then undergoes a sequence of a Diels–Alder reaction with ethylene and heterogeneous AD by Pt/C to form toluene.^81^ More recently, Goldman found that the TD of linear alkanes with ethylene or propylene in a heterogeneous solid–gas system (*i.e.* the combination of a solid molecular pincer-Ir catalyst and gaseous substrates) gives a high yield of α-olefins. The isomerization of a terminal alkene to an internal alkene, to a large extent, could be inhibited. Calculations reveal that the gaseous acceptor effectively captures the Ir dihydride species, which is partly responsible for alkene isomerization.^82^

Catalytic AD reactions under photochemical conditions have also been explored. In general, the photochemical AD proceeds at lower temperatures and offers a higher selectivity for α-olefins in comparison to the thermochemical reactions. An additional advantage is that a hydrogen acceptor is not required in these photochemical reactions. Crabtree reported the first photochemical AD using IrH_2_(PCy_3_)_2_(η^2^-O_2_CCF_3_), giving a TON of seven for the acceptorless dehydrogenation of COA.^83^ Normura and Saito found that RhCl(PMe_3_)_2_CO is a more effective catalyst: the photochemical dehydrogenation of *n*-octane resulted in a turnover frequency (TOF) of 650 h^–1^ at 86 °C.^84^ Goldman applied this Rh catalyst to the photochemical dehydrogenation of cycloalkanes: TONs of up to 5000 with a TOF of 300 h^–1^ were achieved in the run with COA. Mechanistic studies established that the photoextrusion of CO from RhCl(PMe_3_)_2_CO is part of the catalytic cycle.^85^ Using the same catalyst, very recently, the Beller group observed that the added 4,4′-bipyridine could enhance the catalyst stability and light transmittance has a profound effect on the efficiency. Under the optimal light transmittance, the acceptorless dehydrogenation of *n*-octane gives TONs of ∼1500 with 35% selectivity for 1-octene.^86^

### 4.5. Dehydrogenation-based alkane transformation

**Fig. 6:**
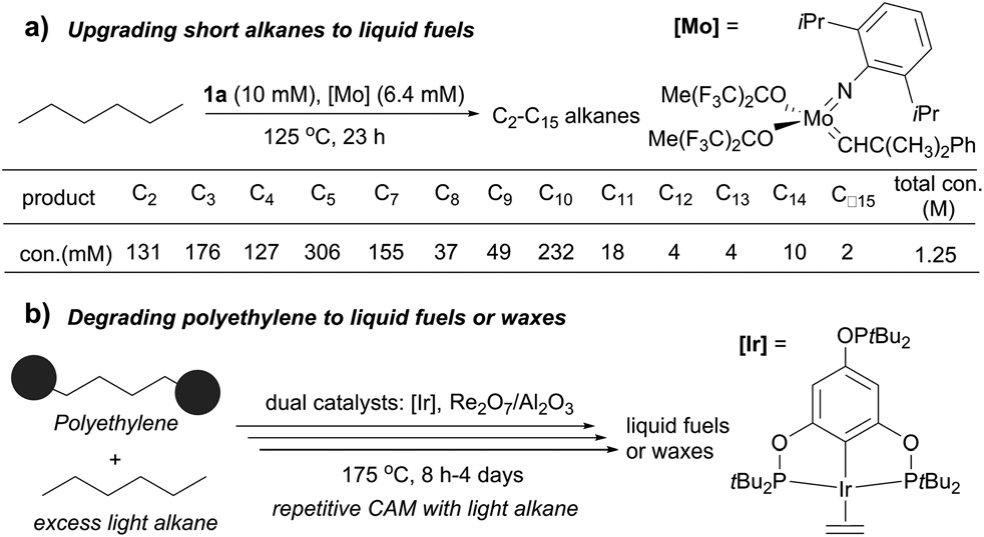
Applications of alkane metathesis.

Several catalytic methods of alkane transformation initiated by AD have been developed during the past decade. Goldman and Brookhart reported a tandem dual catalyst system for alkane metathesis (AM), in which the pincer Ir catalyst (**1a**) effects AD and Schrock catalyst effects olefin metathesis. The process enables the conversion of a linear alkane to linear products, but rapid olefin isomerization prior to metathesis results in the formation of a range of alkanes. For example, the metathesis of *n*-hexane at 125 °C yields C2–C5 and C7–C15 *n*-alkanes (150–200 equiv. relative to Ir) (Fig. 6a). Switching the olefin metathesis catalyst to Re_2_O_7_/γ-Al_2_O_3_, a heterogeneous catalyst, leads to a more robust system, giving TONs of ∼500 for *n*-decane metathesis at 175 °C.^87^,^88^

The Goldman–Brookhart AM system holds promise for upgrading low carbon number *n*-alkanes (*e.g.* C4–C8) to higher molecular weight fuel alkanes. Its reverse process, the degradation of polyethylene (PE) into fuel alkanes using a cross-AM strategy, was recently reported by Huang and Guan (Fig. 6b). Using the Goldman–Brookhart Ir/Re catalyst system, the cross-AM between PE and a light alkane results in the breakdown of a PE chain. Because a large excess of light alkanes is used as the reactant/solvent, the first cross-AM products react further with the light alkanes to give even shorter hydrocarbons. After multiple cycles of cross-AM with light alkanes, the authors demonstrated that HDPE (high density PE) with a molecular weight of up to 1.7 million could be fully degraded to oil and wax. The catalyst system can tolerate commercial HDPE, LDPE and LLDPE that contains phosphinite-based stabilizers and zinc stearate which allowed for the degradation of various post-consumer PE plastics waste.

Bercaw and Labinger devised a novel method, the net coupling of an alkane and a terminal alkene, to upgrade light hydrocarbons into heavier fuel alkanes (eqn (19)).^89^ This system comprises a catalyst for the TD of an alkane and a Ta catalyst (Cp*TaCl_2_(CH_2_=CH_2_)) for the dimerization of the resulting alkene product with another alkene molecule. The subsequent Ir-mediated TD would yield the upgraded alkanes, together with a new alkene for the next cycle of dimerization. Because the Ta catalyst is inactive for the dimerization of internal olefins, the choice of the terminal-selective Ir catalyst (**1b**) is important for this transformation. The slow addition of the terminal alkene, which minimizes the degree of undesired isomerization, is also crucial to the success of the coupling. The authors showed that gradually adding 1-hexene *via* a syringe pump to a mixture of Ir/Ta catalysts in *n*-heptane forms branched C13 and C14 in a 40% total yield (relative to 1-hexene), corresponding to a TON of 60 for **1b**. Despite the low efficiency, the alkane/alkene coupling yields products with controllable selectivity for a desired weight fraction, in contrast to a stochastic product distribution observed for AM.
20

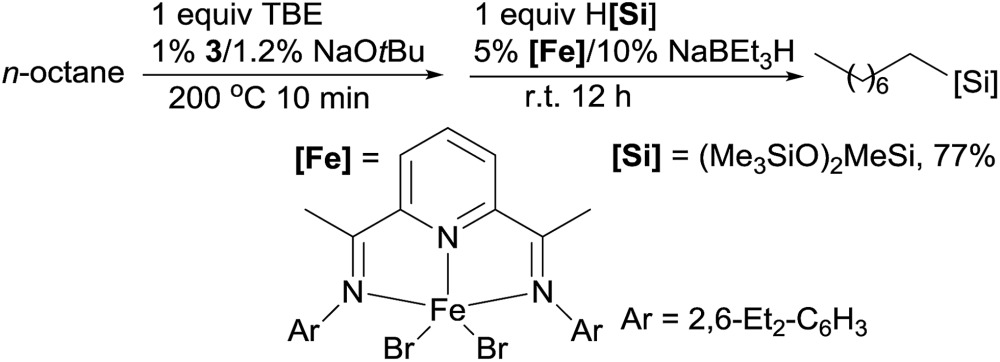




**Fig. 7:**
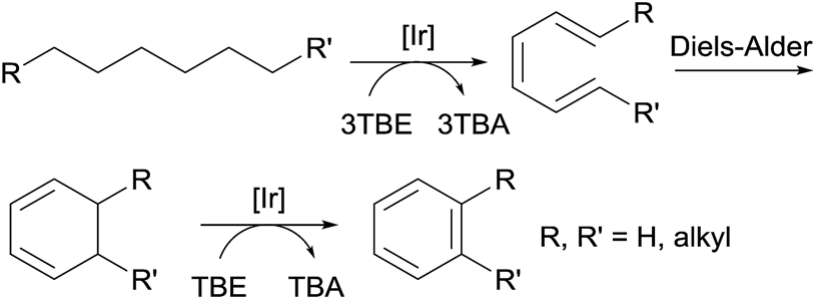
Dehydroaromatization of alkanes.

Aromatic molecules, especially the BTX family of benzene, toluene, and xylene, are valuable feedstocks in the chemical industry. Goldman and Brookhart reported the first example of the homogeneously catalyzed dehydroaromatization of *n*-alkanes to benzene, *n*-alkylarenes or *o*-dialkylarenes (Fig. 7).^80^,^81^,^90^ Using hybrid complex (^iPr4^PCOP)Ir(C_2_H_4_) **4** as the catalyst, the reaction of *n*-octane with four equiv. of TBE affords *o*-xylene as the dominant product, while the reaction of *n*-decane yields four different arenes, with *o*-propyltoluene as the major product.^80^

Another strategy for the synthesis of *n*-alkylarenes was reported by Schrock and Goldman.^91^ The cross-metathesis between an *n*-alkane and ethylbenzene using a combination of the dehydrogenation Ir catalyst **1a** and a thermally stable W-based metathesis catalyst enables the selective formation of *n*-alkylarenes over alkanes.^91^

Linear alkylsilanes are valuable synthetical intermediates in the silicone industry. In 2016, Huang reported the net terminal silylation of alkanes to form linear alkylsilanes using a dual Ir/Fe catalyst system.^92^ The pincer Ir **3** catalysed dehydrogenation gives internal olefins as the major products, which, in the presence of iron catalysts, are isomerized to α-olefins for anti-Markovnikov hydrosilylation. The ineffectiveness of the bis(imino)pyridine Fe complex (**9**) for the hydrosilylation of internal olefins precludes the formation of branched alkylsilanes. For example, the TD of *n*-octane with TBE at 200 °C for 10 min, followed by the isomerization–hydrosilylation with (Me_3_SiO)_2_MeSiH, generates the linear alkylsilane in a good yield (eqn (20)).

## 5. Conclusion and outlook

The period of the last four decades has witnessed significant progress in homogeneously catalyzed alkane transformations through C–H bond functionalisation using methods including radical pathways or carbene/nitrene insertions. In practice, these approaches using highly reactive free radicals or carbene/nitrene intermediates may be primarily applicable to the small-scale production of specialized chemicals, given the use of relatively expensive and/or forcing reagents and inherent regioselectivity. The preference of such methods for the functionalisation of secondary or tertiary C–H over primary C–H bonds renders them more suitable for cyclic substrates, rather than linear alkanes. Moreover, one specific C–H bond of an alkyl chain in a more functionalised molecule may react with a radical or carbene/nitrene with useful selectivity. Thus, one may expect that some transformations, such as C–H fluorination and enantioselective carbene insertion, will find applications in the late-stage labeling of biologically active molecules or in the synthesis of fine chemicals.

From a practical point of view, the functionalisation of the primary sites in simple alkanes is especially attractive for the large-scale synthesis of specialty or commodity chemicals. In this respect, the transition metal-catalysed selective activation of primary C–H bonds holds great potential. The challenge ahead now lies in the development of practical processes that utilize low-cost and environmentally benign reagents. Although highly regioselective, the Rh- or Ru-catalysed alkane borylations using boron reagents are not economically viable. While the formal terminal silylation of alkanes with relatively low-cost silanes using a two-step dehydrogenation–hydrosilylation process has been developed by the author’s group,^92^ the direct silylation of alkanes higher than methane^93^ to form valuable linear alkylsilanes remains to be exploited.

Most important bulk oxygenates contain the functional group at the end of an alkyl chain, therefore, the selective oxidation of alkanes to linear alkyl alcohols, aldehydes, or carboxylic acids is a goal of great interest. Unfortunately, the known methods for alkane oxidation *via* electrophilic C–H activation feature low regioselectivity. An ultimate challenge in this important field is to use molecular oxygen or hydrogen peroxide for selective oxidations, generating water as the only by-product.

Catalytic alkane dehydrogenations typically need a sacrificial hydrogen acceptor to gain high turnover numbers, while the existing systems for acceptorless dehydrogenations suffer from very low efficiency. Another challenge is the poor selectivity for the formation of α-olefins due to the facile isomerization of the kinetic terminal products to internal olefin mixtures. Encouragingly, as noted in the body of this perspective, Goldman and co-workers have found that using practical hydrogen acceptors, ethylene or propylene, can effectively eliminate olefin isomerization, leading to significantly enhanced yields of α-olefins. Thus, further development of long-lived highly efficient catalysts in conjunction with an appropriate hydrogen acceptor and reaction vessel may provide a practical catalytic system for regioselective alkane dehydrogenation. Moreover, efficient dehydrogenation catalysts based on earth-abundant and low-cost base-metals will be a welcome addition.

Regardless of the nature of the different classes of method for alkane functionalisations, catalyst development will continue to be the central theme of research. In-depth mechanistic understanding of the factors controlling reactivity and selectivity will lead to the future development of more efficient catalysts and the discovery of new catalytic reactions. Yet, one must not overlook the power of cooperative catalysis that involves two or more catalysts for new alkane transformations. Moreover, the design of enantioselective catalytic systems will provide a framework for the synthesis of high-value fine chemicals using a simple hydrocarbon as the starting material. One may also expect that developing appropriate catalysts and conditions for photocatalysis and electrocatalysis will be an emerging topic in the area of alkane chemistry.

## Conflicts of interest

There are no conflicts to declare.
